# Glucagonoma and Glucagonoma Syndrome: A Case Report with Review of Recent Advances in Management

**DOI:** 10.1155/2016/1484089

**Published:** 2016-02-14

**Authors:** Ashraf Al-Faouri, Khaled Ajarma, Samer Alghazawi, Sura Al-Rawabdeh, Adnan Zayadeen

**Affiliations:** ^1^Department of General Surgery, King Hussein Medical Center, Amman 11831, Jordan; ^2^Department of Pathology, King Hussein Medical Center, Amman 11831, Jordan; ^3^Department of Radiology, King Hussein Medical Center, Amman 11831, Jordan

## Abstract

The rarity of glucagonoma imposes a challenge with most patients being diagnosed after a long period of treatment for their skin rash (months-years). Awareness of physicians and dermatologists of the characteristic necrolytic migratory erythema often leads to early diagnosis. Early diagnosis of glucagonoma even in the presence of resectable liver metastases may allow curative resection. Herein, we present a typical case of glucagonoma treated at our center and review the literature pertinent to its management.

## 1. Introduction

Since its first description by Becker in 1942 [1], around 300 cases of glucagonoma and glucagonoma syndrome have been described. Consequently, only few surgeons and physicians will be faced with this peculiar diagnosis. Most reported cases of glucagonoma were malignant with many patients presenting with metastatic disease. This highlights the importance of early diagnosis since complete resection of the primary tumor and limited liver metastases offers the only chance of cure.

## 2. Case Presentation

A 64-year-old lady presented to the dermatologist complaining of diffuse erythematous pruritic skin rash of six months' duration. Her clinical history was positive for diabetes for the last 4 years well-controlled by oral hypoglycemic drugs (Glibenclamide 5 mg bid, Metformin 850 mg bid) and hypertension for the last 5 years. She also reported painful tongue and significant weight loss in the last year. Her family history was negative for endocrine disorders.

**Figure 1:**
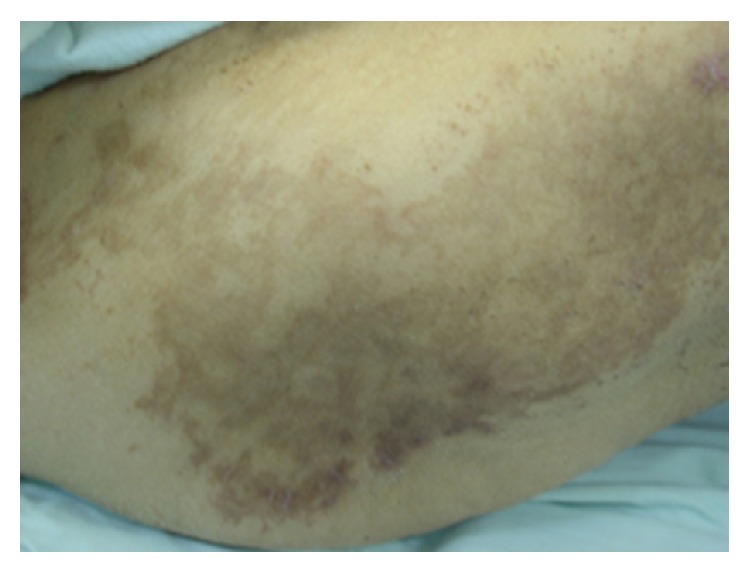
Necrolytic migratory erythema over the back with areas of healing and hyperpigmentation.

**Figure 2:**
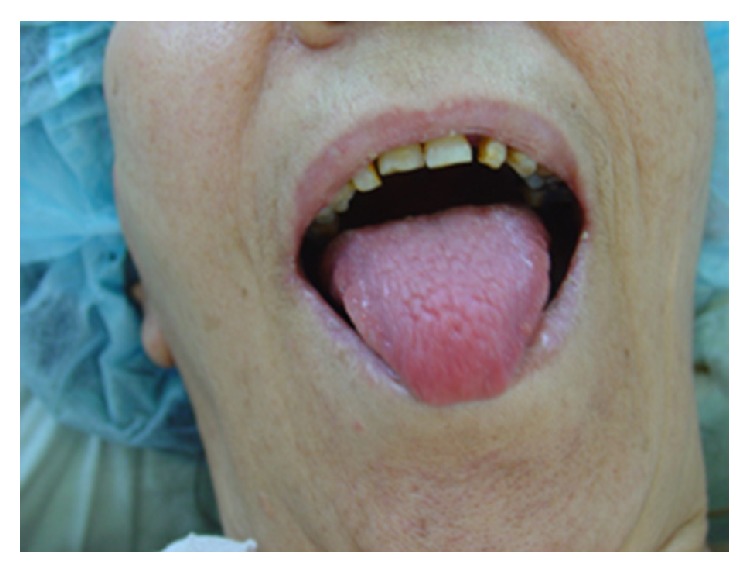
Glossitis.

**Figure 3:**
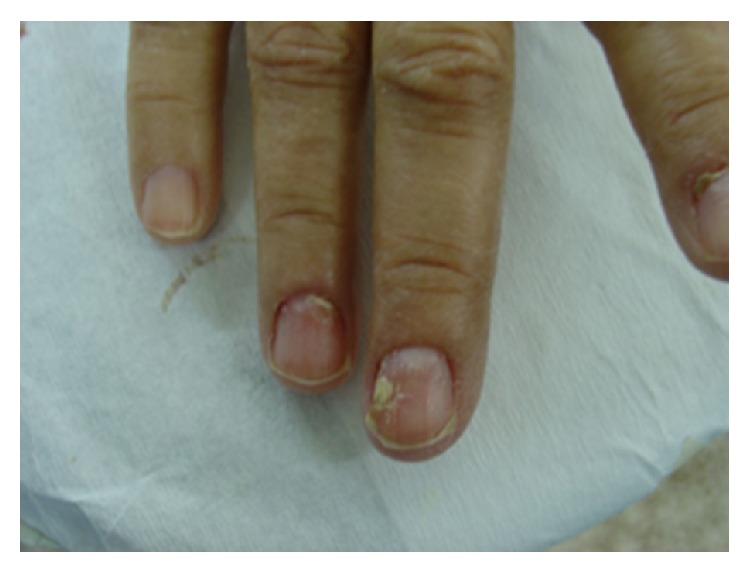
Onychoschizia.

Physical examination revealed an erythematous scaly rash with areas of hyperpigmentation and skin sloughing involving mainly the extremities and lower back (Figure 1). The skin rash tended to occur in crops that later blister and slough while new lesions occur in another area. The lesions were suggestive of necrolytic migratory erythema. Her tongue was atrophic and bright red (Figure 2). Her nails were brittle with longitudinal and transverse fissuring (onychoschizia) (Figure 3).

**Figure 4:**
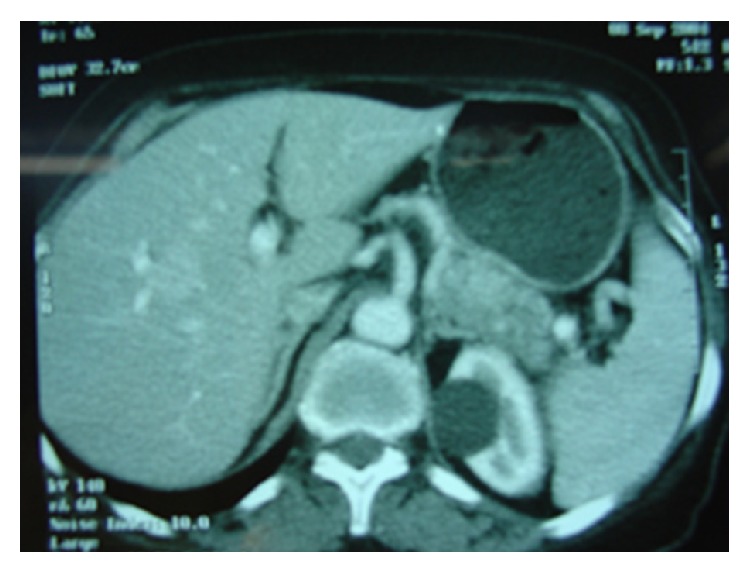
Hypervascular tumor in the tail of pancreas with metastases in segment V of the liver.

Laboratory investigations included blood sugar of 178 mg/dL and glycosylated hemoglobin (HgA1c) of 11.2%. Hemoglobin was 10.2 g/dL and blood film showed microcytic hypochromic anemia. Total protein was 69 g/dL with albumin level of 28 g/dL. Kidney function tests were normal. Abdominal ultrasound revealed a hypoechoic mass in the tail of pancreas with multiple hypoechoic liver lesions suggestive of metastatic disease Accordingly, a contrast enhanced CT scan was done and confirmed the presence of 5 × 5 cm hypervascular lesion in the tail of pancreases with 3 liver metastases (Figure 4). Glucagon level assay was not available at time of presentation.

**Figure 5:**
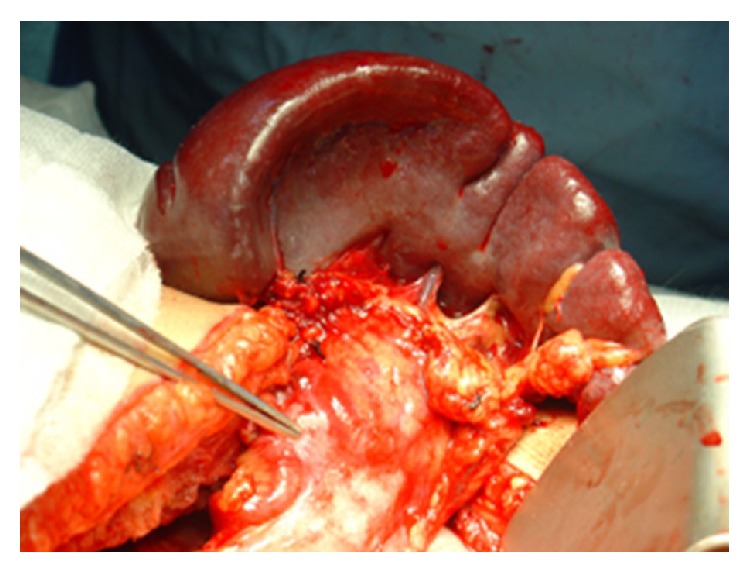
Glucagonoma in the tail of pancreas.

Surgical exploration with a presumptive diagnosis of glucagonoma was thus performed. Distal pancreatectomy with splenectomy as well as wedge resection of a superficial liver lesion was performed (Figure 5).

Histopathological examination confirmed the diagnosis of metastatic pancreatic neuroendocrine tumor which was positive for Chromogranin A, glucagon, and synaptophysin.

The patient fared well postoperatively with disappearance of skin rash with residual areas of hyperpigmentation. She was started on long-acting somatostatin analogue Lanreotide for metastatic disease. Her liver disease remained stable for 2 years of follow-up. Patient died in another hospital 5 years after surgery with disseminated metastatic disease but no autopsy was done.

## 3. Discussion

Glucagonoma is an extremely rare slowly growing, frequently malignant neuroendocrine tumor of the *α*-cells of the pancreas with an estimated incidence of 1/20,000,000/year [2]. It typically presents with glucagonoma syndrome.

The classical description of glucagonoma syndrome includes the characteristic, but nonpathognomonic, necrolytic migratory erythema, new onset of diabetes mellitus, anemia, glossitis, weight loss, neuropsychiatric manifestations, and thromboembolism in the presence of hyperglucagonemia [3]. Apart from NME, other manifestations are quiet common and nonspecific accounting for delay in diagnosis in most cases.

It typically occurs in 6th decade with an age range of 16–88 years reported in the literature [4]. Although earlier studies suggest female predominance (3-4 : 1 female : male), a recent review done by us of 168 cases published in English literature suggests no gender predilection (93 females versus 75 males). Most tumors are sporadic and the minority of patients has MEN-syndrome [5].

Glucagonoma typically occurs in the distal pancreas (≈85% are in body or tail) and is large at time of diagnosis (0.4–25 cm). Most reported cases of glucagonoma were malignant with many patients presenting with metastatic disease (65–75%) [6]. Metastases most commonly occur in the liver followed by peripancreatic lymph nodes.

This highlights the importance of early diagnosis since complete resection of the primary tumor and limiting metastases offers the only chance of cure. The slow growth of the tumor coupled with advances in liver surgery and transplantation may allow curative resection in patients with metastatic disease confined to the liver.

Novel advances in management of metastatic pancreatic neuroendocrine tumors include complex liver resections and liver transplantation [7], percutaneous ablation of liver metastases, long-acting somatostatin analogues, targeted radiotherapy (peptide ligand receptor radionuclide therapy (PRRT) and Radioembolization with Selective Internal Radiation Microspheres [8]), and biologic therapy (Sunitinib and Everolimus). A systematic evaluation of these therapies in management of glucagonoma is impossible due to the rarity of the tumor. Thus, a multimodal approach to management of these rare tumors with individualization for each case is advised [9].
